# Anterograde bile duct drainage for intractable bile leakage after hepatectomy in a patient with previous pancreatoduodenectomy: A case report

**DOI:** 10.1016/j.ijscr.2019.01.017

**Published:** 2019-01-29

**Authors:** Ryohei Murata, Yo Kamiizumi, Chihiro Ishizuka, Sayuri Kashiwakura, Takeshi Tsuji, Hironori Kasai, Yasuhiro Tani, Tsutomu Haneda, Tadashi Yoshida, Koji Ito

**Affiliations:** a Department of Surgery, Iwamizawa Municipal Hospital, 068-8555, Iwamizawa-shi, Japan; b Department of Gastroenterological Surgery I, Hokkaido University Graduate School of Medicine, 060-8648, Sapporo-shi, Japan

**Keywords:** Bile leakage, Hepatectomy, Anterograde drainage, Pancreatoduodenectomy, Choledochojejunostomy

## Abstract

**Introduction:**

Endoscopic retrograde drainage is effective for managing bile leakage, which is relatively common after hepatectomy without bile duct reconstruction. However, the procedure is difficult to perform after pancreatoduodenectomy with choledochojejunostomy. We present a case of anterograde bile duct drainage for intractable bile leakage after hepatectomy in a patient with previous pancreatoduodenectomy.

**Presentation of case:**

An 80-year-old woman with a history of pancreatoduodenectomy for distal biliary cancer and adjuvant chemotherapy presented with bile leakage. Six years after the pancreatoduodenectomy, she underwent partial hepatectomy for suspected metastasis or intrahepatic cholangiocarcinoma. On the 9th postoperative day, bile leaked from her drainage tube forming an abscess cavity; this continued until the 28th postoperative day. We attempted selective anterograde drainage from the cut surface of the liver under fluoroscopic guidance using a guidewire and Cobra-type catheter. We selectively cannulated the entrance hole of the bile duct. Twenty days after the drainage, the abscess cavity disappeared. After 41 days, the tube was removed, and the patient was discharged. We suggest this procedure as a possible treatment option for difficult bile leakage cases.

**Discussion:**

A case of intractable bile leakage after hepatectomy in a patient with a previous history of pancreatoduodenectomy is difficult to manage, and usually needs surgical intervention. The effect of selective cannulation of the entrance hole of the bile duct has not been studied.

**Conclusion:**

Selective anterograde bile duct drainage for intractable bile leakage after hepatectomy in a patient with a previous history of pancreatoduodenectomy successfully resolved bile duct leakage in our patient.

## Highlights

• Endoscopic retrograde drainage is effective for managing bile leakage.
• Bile drainage after pancreatoduodenectomy with choledochojejunostomy is difficult.
• Post-hepatectomy bile leakage in a patient with prior pancreatoduodenectomy.
• Selective anterograde bile duct drainage was successful in treating the condition.

## 1 Introduction

Bile leakage is a relatively common postoperative complication after hepatobiliary surgery. Postoperative bile leakage is reported to occur in 0.8–1.4% and 3.6–12% of patients undergoing laparoscopic cholecystectomy (LC) and hepatectomy without bile duct reconstruction, respectively [1,2]. Endoscopic retrograde drainage is a common approach for managing bile leakage, but it is difficult to perform if a patient has a history of choledochojejunostomy. As the approach to the bile duct via the choledochojejunostomy point is very long and winding, endoscopic drainage is not suitable and surgical intervention is required in many cases. In this case report, we present a unique technique for a less invasive treatment for bile leakage in post-hepatectomy patients with a previous pancreatoduodenectomy. This work has been reported in line with the SCARE criteria [3].

## 2 Presentation of case

An 80-year-old woman had a history of pancreatoduodenectomy for distal biliary cancer and had undergone adjuvant chemotherapy. Six years after the pancreatoduodenectomy, she underwent partial hepatectomy of the S4/8 mass for suspected metastasis or intrahepatic cholangiocarcinoma.

**Fig. 1:**
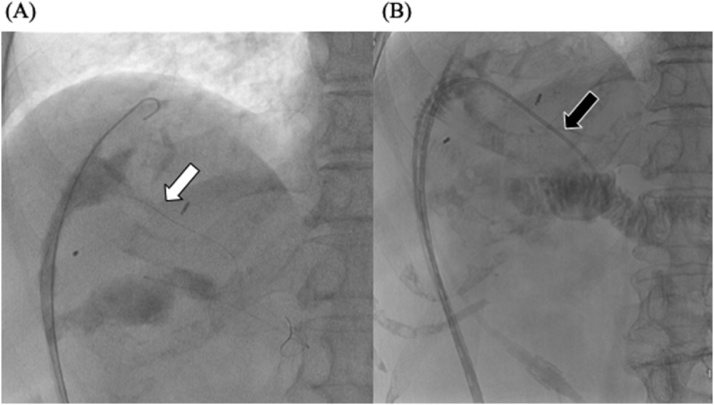
X-ray fluoroscopic examination on the 28^th^ postoperative day, showing (A) the flow to the anterior branch of the right intrahepatic bile duct and the cannulated guide wire (white arrow). (B) The guide wire was advanced toward the jejunum via the junction of choledochojejunostomy under the assistance of 5Fr. Cobra-type catheter (black arrow).

**Fig. 2:**
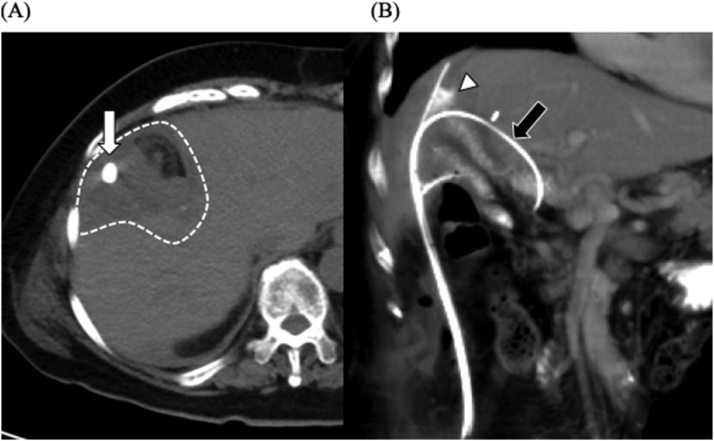
(A) Enhanced computed tomography (CT) on the 9^th^ postoperative day. The abscess cavity had formed around the surface of the hepatectomy from the bile duct injury point (dashed line), showing the indwelling drainage tube (white arrow). (B) Enhanced CT on the 20^th^ day after the intervention, showing the Cobra catheter in the bile duct (black arrow). The abscess cavity had almost disappeared (white arrowhead).

**Fig. 3:**
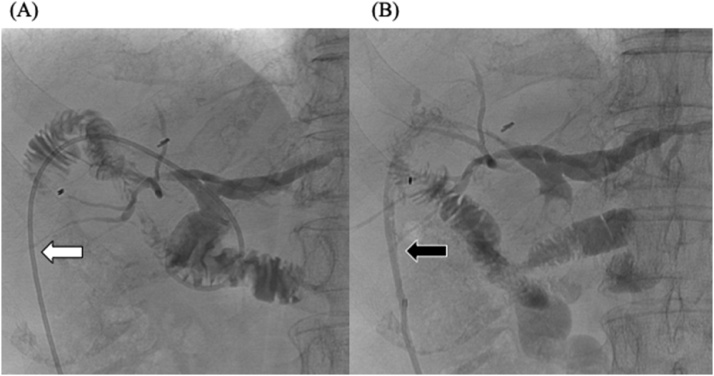
X-ray fluoroscopic examination on the 41st postoperative day. (A) The catheter was in the branch of the right intrahepatic bile duct (white arrow), and no leakage was detected. (B) The external fistula was completely formed (black arrow), and there was no abscess.

Nine days after the second surgery, bile leakage from the indwelling drainage tube occurred, forming an abscess cavity (Fig. 1). Laboratory tests of the drain effluent revealed the following: total bilirubin (T-Bil) 13.3 mg/dl, direct bilirubin (D-Bil) 10.0 mg/dl, and amylase (AMY) 2396 IU/l, and blood tests revealed the following: T-Bil 1.0 mg/dl, D-Bil 0.4 mg/dl, and AMY 20 IU/l. We changed the tube to wash the cavity and continued saline irrigation of the abscess cavity; however, the bile leakage continued until the 28th postoperative day. It was difficult to perform retrograde drainage of the bile by endoscopy because of the history of pancreatoduodenectomy with choledochojejunostomy. Contrast-enhanced radiography of the cavity revealed flow to the anterior branch of the right intrahepatic bile duct. We selectively cannulated the entrance hole of the bile duct with a 0.035-inch hydrophilic guide wire and a 5 Fr Cobra-type catheter, which is usually used for angiography. An indwelling drainage tube was inserted from the junction of the pancreatoduodenectomy to outside the body, via the abscess cavity (Fig. 1). We also placed a catheter to drain the content of the abscess cavity. Plane computed tomography (CT) showed that the cavity had reduced in size and almost disappeared 20 days after the intervention (Fig. 2). Forty-one days after the intervention, when the external fistula was formed, we removed the tube (Fig. 3). The patient was discharged 4 days later, and there was no recurrence of bile leakage thereafter.

## 3 Discussion

Bile leakage is a critical complication after hepatectomy, which causes biliary peritonitis and may sometimes lead to severe consequences. Postoperative bile leakage is defined by the International Study Group of Liver Surgery (ISGLS) as a “bilirubin concentration in the drain fluid at least three times the serum bilirubin concentration on or after postoperative day 3” or as “the need for radiologic or operative intervention resulting from biliary collections or bile peritonitis” [1]. They also suggest grading the severity of the bile leakage into three stages, from A to C. Grade A bile leakage causes no change in a patient’s clinical management, Grade B bile leakage requires active therapeutic intervention but is manageable without relaparotomy, and Grade C bile leakage requires relaparotomy [1]. Our case could be considered Grade C because it was impossible to treat following the usual procedure.

Previously, bile leakage used to require surgical intervention. However, retrograde endoscopic drainage is efficient and is increasingly being used to manage bile leakage [4,5]· The success rate of the endoscopic therapy for bile leakage is 87–100% after LC and 75% after hepatectomy [4,5]. The success rate of the endoscopic treatment for bile leakage after hepatectomy depends on whether the stent covers the leakage point or not (83% vs. 43%), but it is sometimes difficult to bridge the stent to the distal bile duct leakage point [4]. Bile leakage is divided into two types: one in which bile leaks into the gastric tract and the other in which there is no bile leakage into the gastric tract. Endoscopic therapy and percutaneous drainage are effective for the former type, but they are not applicable to the latter type, which needs reconstruction of the bile duct or eradication of bile excretion. Some of the methods of eradication reported are ethanol ablation [6], *N*-butyl cyanoacrylate occlusion [7], and portal embolization [8]. In our case, there was an injury on the surface due to hepatectomy that was difficult to stent to cover the leakage point. In this case the retrograde approach was impossible, and thus we attempted a unique intervention technique involving selective anterograde drainage to overcome the existing difficulties.

We decided to cannulate the leakage point on the surface that was injured during hepatectomy. Although this may be considered reckless, we were able to cannulate the wire and catheter to the small leakage point. We thought that the bile in the abscess might destroy the tissue in the cavity to form a mortar-shaped leakage point. Such a leakage point is formed after a considerable period of time, around two weeks or more, if the leakage is left untreated. It is essential that the surgeon or radiologist has the requisite skills to control the wire and catheter for anterograde drainage. We tried to set the tube to make a fistula outside the jejunum. The tube was removed 41 days after cannulation after confirming that the cavity had disappeared. Our technique was successful in treating the patient, for whom the retrograde approach could not be used.

## 4 Conclusion

We report a case wherein the technique of selective anterograde bile duct drainage for intractable bile leakage after hepatectomy in a patient with a previous history of pancreatoduodenectomy successfully resolved bile duct leakage in the patient. The technique described herein could be considered an option for managing intractable bile leakage non-operatively, or before surgical intervention.

## Conflict of interest

None of the authors has any conflict of interest to declare.

## Funding sources

This report did not receive any specific grant from funding agencies in the public, commercial, or not-for-profit sectors.

## Ethical approval

Ethical approval has been exempted by our institution.

## Consent

Written informed consent was obtained from the patient for the publication of this case report and accompanying images. A copy of the written consent is available and can be reproduced whenever needed.

## Author contributions

RM acquired and interpreted the data and drafted the manuscript. YK performed the operation and perioperative management of the patient. CI, SK, TT, HK, YT, TH, TY, and KI participated in the operation, perioperative management of the patient, and revision of the manuscript. All authors read and approved the final manuscript.

## Registration of research studies

None.

## Guarantor

Ryohei Murata, Yo Kamiizumi.

## Provenance and peer review

Not commissioned, externally peer-reviewed.
